# The methodology of the Agile Nudge University

**DOI:** 10.3389/frhs.2023.1212787

**Published:** 2023-11-29

**Authors:** Jade Mehta, Christopher Williams, Richard J. Holden, Britain Taylor, Nicole R. Fowler, Malaz Boustani

**Affiliations:** ^1^ Center for Health Innovation and Implementation Science, School of Medicine, Indiana University, Indianapolis, IN, United States; ^2^ Sandra Eskenazi Center for Brain Care Innovation, Eskenazi Health, Indianapolis, IN, United States; ^3^ Department of Health and Wellness Design, School of Public Health - Bloomington, Indiana University, Bloomington, IN, United States; ^4^ Department of Medicine, School of Medicine, Indiana University, Indianapolis, IN, United States; ^5^ Center for Aging Research, Regenstrief Institute, Inc, Indianapolis, IN, United States

**Keywords:** research training, Alzheimer's disease, behavioral science, implementation science, Agile science

## Abstract

**Introduction:**

The Agile Nudge University is a National Institute on Aging-funded initiative to engineer a diverse, interdisciplinary network of scientists trained in Agile processes.

**Methods:**

Members of the network are trained and mentored in rapid, iterative, and adaptive problem-solving techniques to develop, implement, and disseminate evidence-based nudges capable of addressing health disparities and improving the care of people living with Alzheimer's disease and other related dementias (ADRD).

**Results:**

Each Agile Nudge University cohort completes a year-long online program, biweekly coaching and mentoring sessions, monthly group-based problem-solving sessions, and receives access to a five-day Bootcamp and the Agile Nudge Resource Library.

**Discussion:**

The Agile Nudge University is evaluated through participant feedback, competency surveys, and tracking of the funding, research awards, and promotions of participating scholars. The Agile Nudge University is compounding national innovation efforts in overcoming the gaps in the ADRD discovery-to-delivery translational cycle.

## 1. Introduction

Millions of Americans are living with Alzheimer's Disease and other Related Dementias (ADRD) with an economic burden exceeding one trillion dollars (1–6). The cognitive, financial, and social detriments of ADRD disproportionally impact minority groups (7–9). African American individuals are 2–4 times more likely to develop ADRD than their white counterparts yet are 35% less likely to receive an ADRD diagnosis. Disparity in ADRD care contributes to rising differences in life expectancy between urban and rural communities (10).

Despite heavy intellectual and financial investments by the National Institutes of Health (NIH), the individual, familial, and societal burdens of ADRD have continued to rise due to the limited scalability and implementability of discoveries (1–3, 11). Successful implementation and dissemination of healthcare discoveries requires changing the behaviors of patients, family caregivers, clinicians, healthcare administrators, and others interacting within various complex adaptive healthcare delivery organizations (1–3). Appropriate behavioral changes are essential to overcoming existing health disparities, suboptimal quality of care, and mitigating poor health outcomes (11–24).

**Figure 1:**
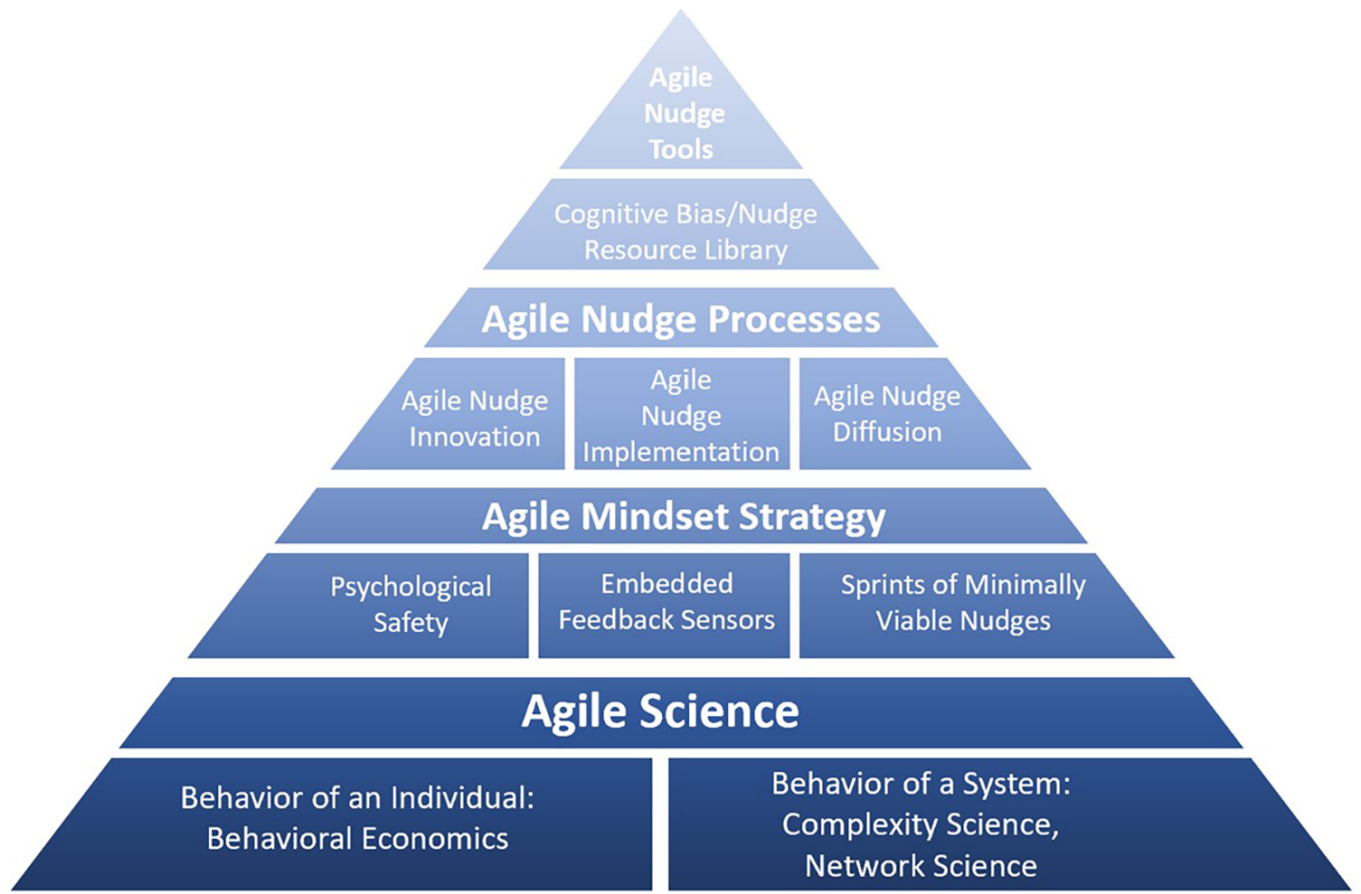
Conceptual model the of theoretical foundation for the Agile Nudge University.

Since 2008, Indiana University has offered bootcamps, a graduate certificate course, and other research training programs in the science of innovation and implementation with diverse cohorts of learners (60% female, 30% from under-represented minority groups) (25). Leveraging past research training successes in translation science fields, scientists at Indiana University, with support from the NIH's National Institute on Aging (NIA), developed the Agile Nudge University to strengthen the discovery-to-delivery pipeline in applied ADRD research (11, 26–35). The program is offered as an open-source research training platform for the scientific community. The Agile Nudge University provides training and coaching in rapid, iterative, and adaptive problem-solving techniques for scientists on how to design, implement, and diffuse evidence-based behavioral and social science interventions, or “nudges,” to address health disparities and improve the care of people living with ADRD (11, 26–33, 36–42). The program was built on the foundation of Agile Science that aims to understand, predict, and steer the behavior of individuals and social organizations (11, 12, 26, 37–42). The short-term aims of the Agile Nudge University are to help participants develop and practice skills in Agile Nudge Innovation, Agile Nudge Implementation, and Agile Nudge Diffusion (13–18, 43–56). The long-term aim of the Agile Nudge University is to build a racially, ethnically, and geographically diverse network of highly engaged ADRD scholars able to skillfully apply Agile Science to create a sustained, evidence-based, impact on the nationwide delivery of clinical and community based ADRD care (13–18, 43–56). This paper describes the methodological tools, processes, and strategies developed for the Agile Nudge University (See Figure 1).

## 2. Methodology

The Agile Nudge University is comprised of core faculty, a program manager, a communication coordinator, an education services coordinator, research consultants, and lecturers, with an internal steering committee and an advisory board. Core faculty are responsible for knowledge acquisition of the science and processes taught as well as providing personalized coaching. Core faculty are involved in 2 of the 3 fields: ADRD research, Agile methods of innovation and implementation, and behavioral and social science research. Lecturers have expertise in at least one of the three fields. The Agile Nudge University recruits, trains, mentors, and supports ADRD scientists to become experts in Agile Science and its methodology. The minimum eligibility criteria for participants are having a college degree and being interested in conducting research in the behavioral and social sciences for ADRD. Nudge University scholars are expected to range from graduate students to senior PhD scientists to established medical professionals. Disciplines of interest include epidemiology, biostatistics, nursing, pharmacy, medicine, psychology, economics, health policy, and behavioral sciences. To address the need for equal access in ADRD care and innovation, scientists of underrepresented minority groups, women, and disabled persons are of specific interest.

**Figure 2:**
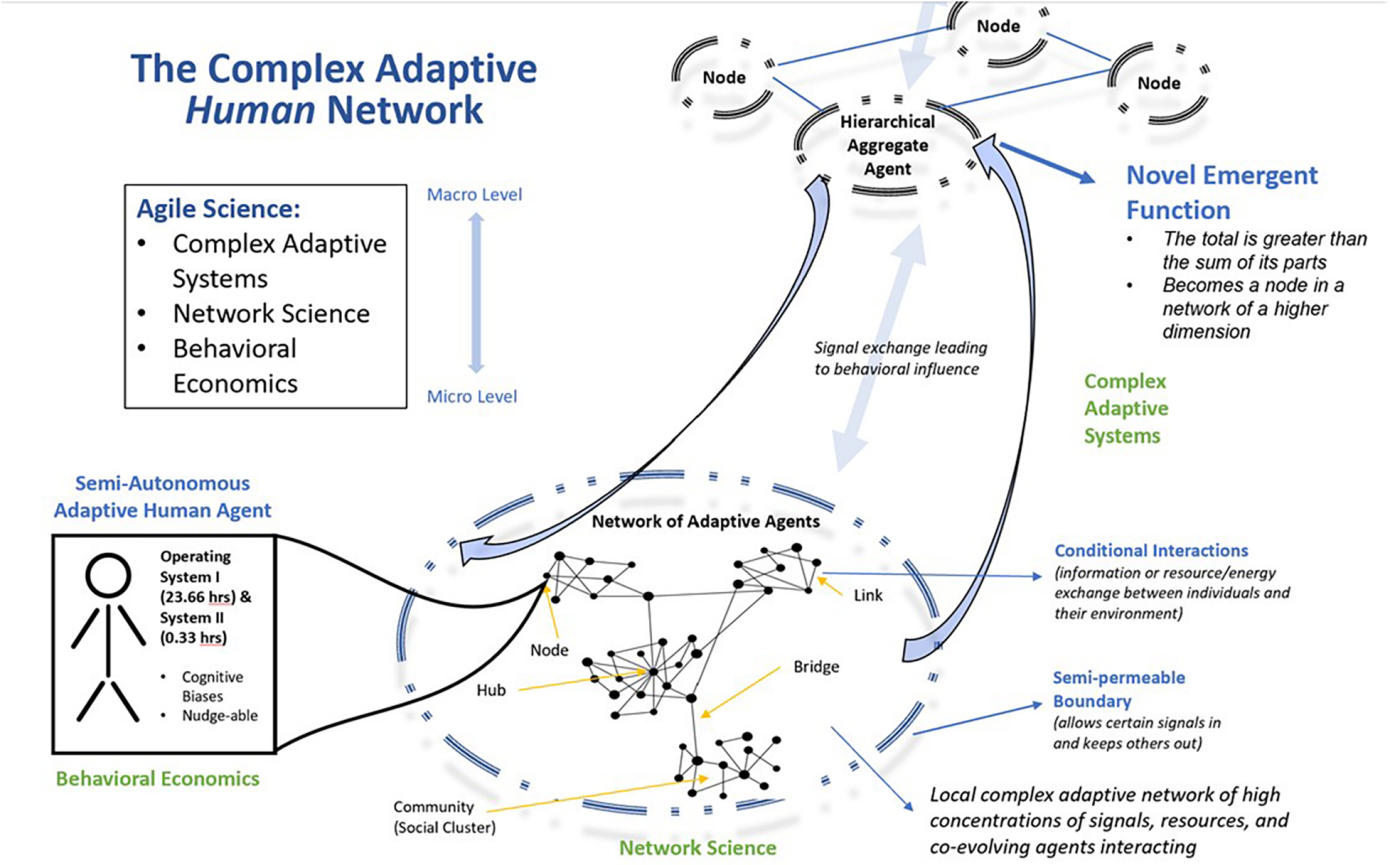
Conceptual model of a complex adaptive human network.

The tools, processes, and strategies developed by the Agile Nudge University are guided by Agile Science. Agile Science combines insights from behavioral economics, complexity science, and network science to model healthcare delivery systems as dynamic evolving networks of numerous interconnected, semi-autonomous, individual human agents (See Figure 2). Agents are contained within a semipermeable boundary that filters the flow of information and energy exchanges with their surrounding environment (11, 12, 26, 55–57). Thus, implementing or diffusing a new discovery in a dynamic and evolving human network requires accounting for temporal and hierarchical variation within the network as well as its external sociocultural contexts (39, 58).

*Behavioral economics* is a discipline that seeks to explain human decision-making, generally using the dual-processing model in which “System 1” is synonymous with a fast and intuitive form of cognition, whereas “System 2” is its slow and deliberate counterpart (36, 59, 60). Due to the mental resource constraints of “System 2” processing, “System 1” is hypothesized to be far more dominant, but its operation produces cognitive tendencies that can result in decisions that differ from a deliberate, comprehensive situation assessment (6, 7, 36, 60). Nudges can be defined as any modification in the physical, social, or digital environment that encourage certain behaviors without forbidding choices (36, 37). Nudges engineer the environment to leverage cognitive tendencies and steer behavior towards pro-social goals (27). Nudges have been found to be effective tools to reduce health disparities, increase guideline adherence, decrease caregiver burden, and optimize care with estimated success rates ranging from 62% to 73% (61–67). Examples of nudges in healthcare contexts range from changing default prescription settings to peer comparison letters sent to clinicians to smart-watch reminder apps for ADRD patients (65–67).

*Complexity science* describes a complex adaptive system as an open, dynamic, flexible network of numerous interconnected members who act in nonlinear ways, are continually co-evolving with their surrounding environment, and are constantly exchanging information or resources (58, 68–72). Complex adaptive systems have a structured hierarchy of energy distribution and information exchange, allowing their patterns of activity to be predictable (72). *Network sciences* map such systems with individual human agents being nodes, highly connected nodes being hubs, the path between nodes being links, aggregations of nodes as communities, and special links connecting two otherwise separate communities as bridges (58, 68–71). The quantifiable aspects of a network include the number of nodes within a network, the number of connections a node has or the degree, the total number of links in a network or sum of all degrees, the probability distribution of node degrees, and the clustering coefficient which measures a network's density of links and can be calculated locally or globally (58, 68–71). The hub is the most highly connected node within a network, and adoption of a belief or behavior by the hub provides the tipping point for creating a social norm (58, 68–71). By identifying the interconnection of each of these components, minimally viable nudges implemented in communities with low resistance and high social proof can lead to participation by the hub and network-wide social contagion (72, 73).

Accounting for the heuristic-driven nature of individual humans each with competing priorities and interacting in a network of energy and resource constraints, Agile Science can inform project management strategies to increase efficiency and reliability (27, 37, 55–60). The Agile mindset strategy guides the development of psychologically safe cultures; the design and embedment of sensors within both the internal and external environment of the network; and the initiation of rapid and iterative testing (sprints) of minimally viable nudges (27, 60). Delineating time and space for a psychologically safe team culture prioritizes collaboration to increase the accuracy of information exchange between members, where all members are communicative of failure and comfortable giving or receiving honest feedback (27). Not prioritizing psychological safety can erode trust, negatively impacting interpersonal communication, team performance, and care delivery (74–79). Embedding “sensors” requires investing time and space to build appropriate timely, actionable, and nonjudgmental feedback loops in a team-based setting as well as listening for outside rumors and gossip (27, 68–71). Monitoring communication channels is critical for deciphering signals and noise within a complex adaptive human network to gauge information exchange between nodes, hubs, and local communities (68–71). Upon securing support and feedback, sprints are cycles of rapid solution testing and modification selected to promote agility for the design, implementation, or diffusion of minimally specified prototypes (11, 26, 36–39). By engineering environments that prioritize agility, research teams are better equipped to scale and sustain evidence-based behavioral and social science interventions within diverse social organizations (11, 26, 27, 36–39).

**Figure 3:**
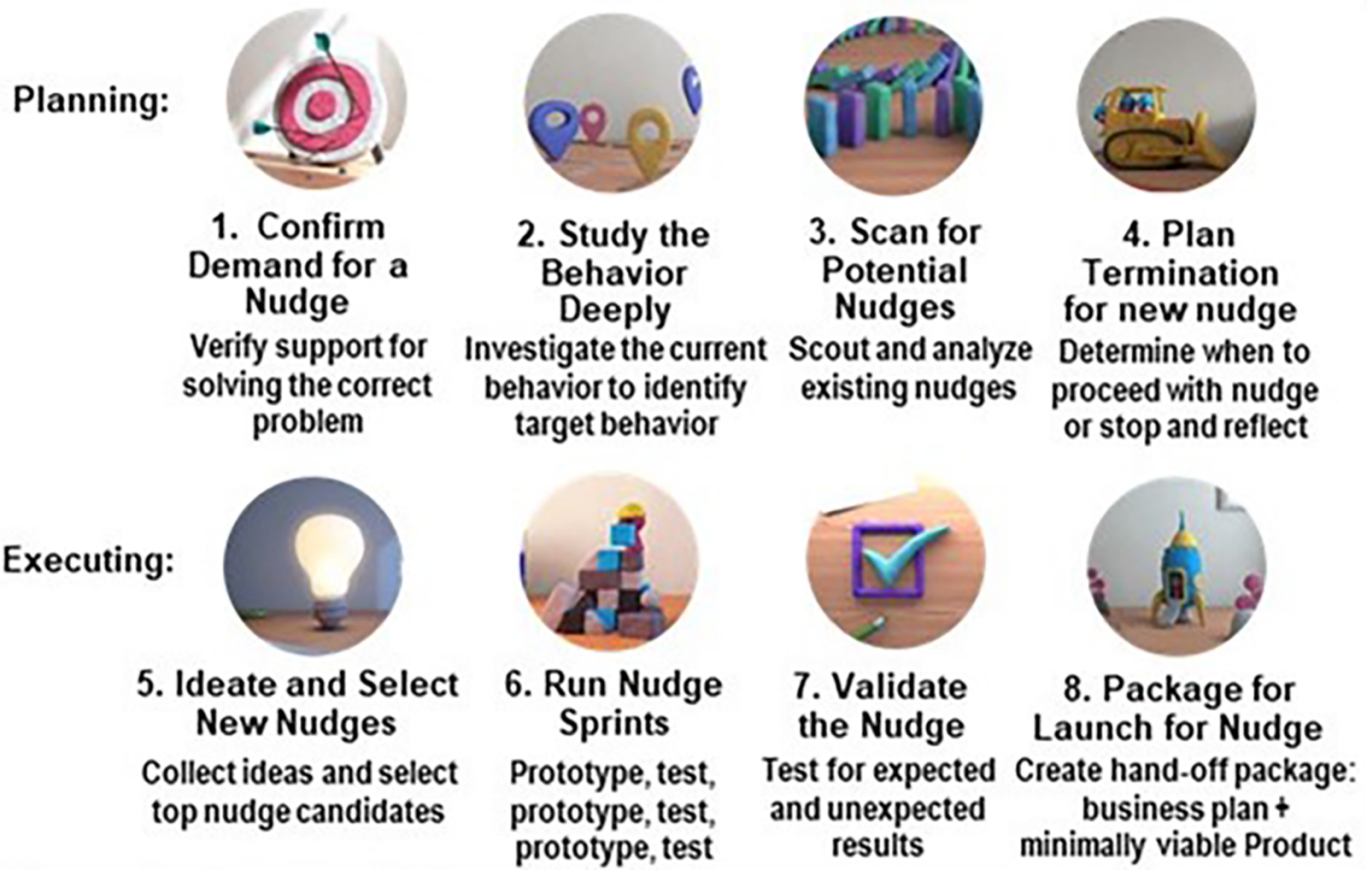
The 8 steps of Agile Nudge Innovation.

Agile Nudge Innovation, Agile Nudge Implementation, and Agile Nudge Diffusion processes operationalize Agile Science to create widespread evidence-based change within the healthcare delivery system (11, 12, 26, 36–39). Agile Nudge Innovation is used when there are no existing evidence-based minimally viable nudges and a new one must be developed (26, 37). The first four steps of Agile Innovation are the planning stages: confirm demand for a nudge, study the behavior deeply, scan for existing nudges, and create a termination plan for any nudge (see Figure 3). The latter 4 steps of the process consist of execution in parallel construction: ideate and select top nudge candidates, run nudge sprints, validate the nudge, and create a business package with minimum nudge specifications.

**Figure 4:**
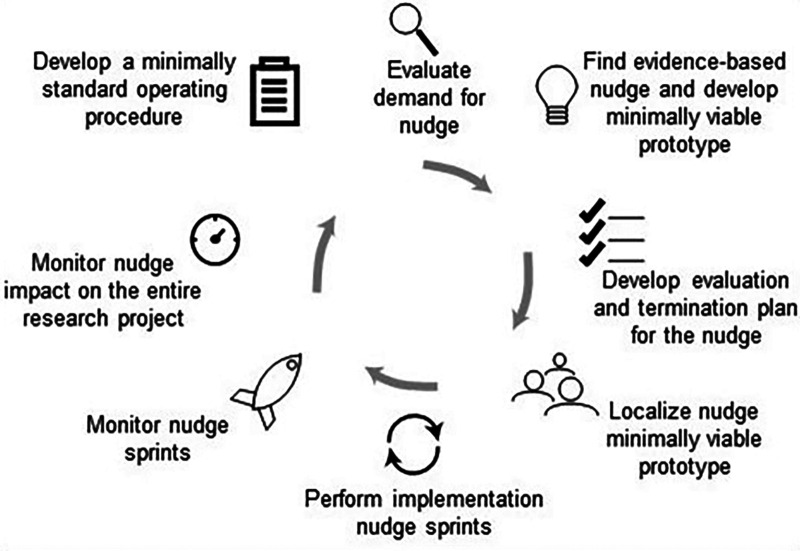
The 8 steps of Agile Nudge Implementation.

If there is already an evidence-based available nudge to a problem, Agile Nudge Implementation can be employed (12, 26, 37, 39, 40). The 8 steps of Agile Nudge Implementation use decades of projects and interventions to optimize the successful and sustainable implementation of nudges (12, 26, 37–39, 43–46). The steps include evaluating demand for the nudge, identifying an evidence-based nudge, developing evaluation and termination plans, assembling a team to develop the minimally viable nudge prototype, performing implementation sprints, monitoring nudge performance, assessing whole system performance, and creating a minimally standardized operation procedure for the nudge implementation (see Figure 4).

**Figure 5:**
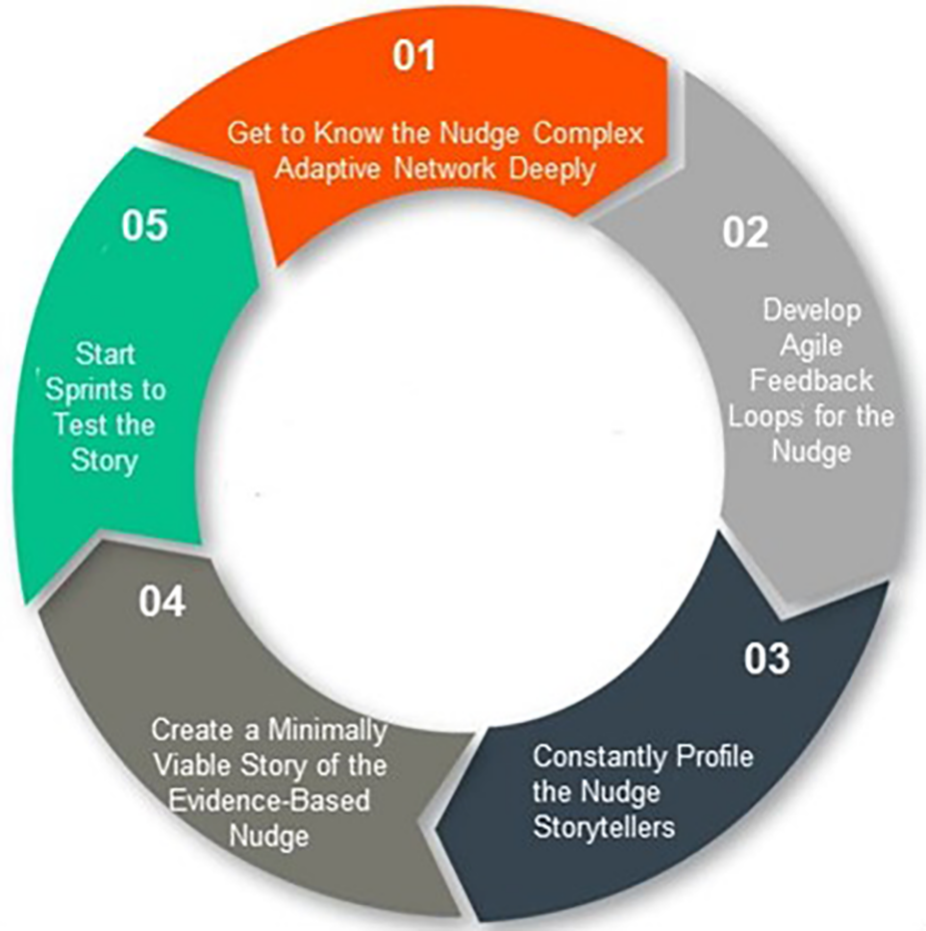
The 5 steps of Agile Nudge Diffusion.

Finally, Agile Nudge Diffusion operationalizes the principles of complexity and network sciences to rapidly disseminate behavioral change throughout a social organization to widen the reach of an evidence-based nudge intervention (26). Agile Nudge Diffusion requires getting to know the complex adaptive human network deeply, developing feedback loops, profiling storytellers, creating a story for the nudge implementation, and running sprints to test the effectiveness of that story on target populations for dissemination (see Figure 5). By mapping the hubs, bridges, degrees, probability distribution, and clustering coefficient of a network, one can identify communities most likely to adopt and spread the nudge while using storytelling to appeal to cognitive biases and create demand (58–61, 68–71, 80). The combination of the 3 Agile nudge processes allows for the creation, sustenance, and dissemination of evidence-based nudges which can be targeted for improving ADRD care and reducing subsequent health disparities.

To aid in nudge innovation, implementation, and diffusion, tools developed for the Agile Nudge University will be made publicly available in an online resource library free of charge to an unlimited number of scientists internationally. The resource library will consist of a catalogue of cognitive heuristics, evidence-based nudges, and ADRD scholar projects.

## 3. Results

Our interdisciplinary team of scientists at Indiana University used insights from Agile Science to develop the NIA funded Agile Nudge University program (# R25AG078136). The Agile Nudge University consists of annual five-day Bootcamps, biweekly personalized coaching and mentorship sessions, monthly Innovation Forums for group-based problem-solving, and an online resource library. Thus, the Agile Nudge University combines hands-on training and feedback with self-driven learning, research, and deliberate practice under expert mentorship. This design fulfills the objectives of the NIH Health Disparities Research Framework to address environmental, sociocultural, behavioral, and biological health disparities related to aging (81). All research training activities are conducted through an ADA-compliant secure Zoom web platform and adapted to each scholar's unique strengths and interests. This format effectively blends teaching and coaching for knowledge acquisition and skills development as well as deliberate practice with timely, actionable, and nonjudgmental feedback. Scholars are also required to complete a Responsible Conduct in Research module, addressing the role of clinicians in ADRD research, handling protected health information, conflict of interests, and research codes of ethics. Similarly, participants complete an online module examining the impact of ageism, sexism, and racism on research and the treatment older adults receive. ADRD scientists are then asked to identify examples of these constructs and explore how to develop nudge research projects which address system-wide factors.

The resource library can be found on the IU-Center for Health Innovation and Implementation Science Agile Nudge University program page (www.hii.iu.edu). The library provides a catalogue of cognitive heuristics, sorting them into categories with descriptions of how they affect human decision making, each with a power rating (82). The resource library also provides a catalogue of evidence-based nudges to increase the efficiency of searching for evidence-based nudges matched for specific digital, physical, or social environments. Each nudge described has associated cognitive biases, problem and target behaviors for intervention, a description for replication, and the effect size of the specified impact (82). The resource library also supplies the Agile Nudge University Toolkit which gives step-by-step instructions for designing and implementing selected nudges (82). After each cohort of the Agile Nudge University, the resource library will be updated to include all Innovation Forum generated solutions and experiential case studies of Agile Science, Agile mindset, and Agile nudge processes.

The monthly group-based problem-solving sessions are 2-hours long and structured with the minimum specification of Innovation Forums to proactively address shortcomings in the design, implementation, or diffusion of ADRD-related nudges (see Table 1). Innovation Forums identify problems, gather key stakeholders, and generate a high volume of creative solutions to effectively solve complex issues working with the impacted populations (83). Innovation Forums require a forum coordinator, a subject-matter expert speaker, an administrative coordinator, a solution tracker, and a facilitator who enforces the ground rules. The activity starts with 15 min of time and space for networking, then a speaker presents the challenge for 10 min, 5 min of clarifying questions are offered, followed by 45 min of discussion and solution generation, and a closing 15 min of informal networking and discussion. Everyone who participates must provide at least one original solution and, to promote a positive environment, critiquing other's ideas is prohibited. Solutions are generated under the assumption that there are no significant resource constraints to maximize innovative thinking.

**Table 1:** Innovation forum minimally standardized operating procedure.

Innovation forums	
Team member	Function
Forum Coordinator	The primary organizer of the event; responsible for ongoing monitoring and evaluation of the event as well as maintaining communication with the presenter on any forum-related needs or preferences.
Presenter	Owns the challenge and is responsible for identifying a small group of individuals to whom a personal invitation will be sent.
Administrative Coordinator	Provides logistical and administrative support throughout planning process and during the event.
Solution Tracker	Takes and distributes notes during Innovation Forum planning meetings and records solutions during the day of the event.
Facilitator	Conducts the Innovation Form, ensures smooth knowledge transfer between presenter and audience, and profiles and engages the audience. The facilitator is not a content expert, but rather promotes conversation, clarity, and understanding.
Activity and time	Description
Opening 15 Min of Networking	Allows time and space for attendees to connect.
10 Min for Presentation of the Challenge	Reserved for the identified speaker to present their implementation or delivery challenge; the speaker may use whatever visual aids they feels are necessary (PowerPoint, handouts, etc.).
5 Min of Clarifying Questions from the Audience	Utilized to clarify anything within the scope of the presentation. The Facilitator must ensure there are no solutions generated during this time and the Facilitator also advocate that the called-upon person state his or her concern in question form.
45 Min of Discussion and Solution Generation	Used for generating solutions, additional questions, and general brainstorming.
Closing 15 Min Of Networking	Intended To Provide Closure To The Discussion In A Less Structured Manner.

The first two days of each virtual Bootcamps focus on knowledge acquisition with the remaining days used for skill development (see Table 2). Each day different ADRD case examples are examined. The end of each session includes time and space for group-based debriefing. The first day covers Agile Science, the Agile mindset, and how behavioral economics and choice architecture play into the ADRD setting. The second day provides an in-depth focus on Agile Nudge Innovation, Implementation, and Diffusion processes using ADRD case studies within primary care, specialty care, and community-based settings. The rest of the days include self-paced online interactive simulations of the Agile processes in designing, implementing, or diffusing nudges for ADRD in various settings. Employment of simulation training in the Agile Nudge University Bootcamps is integral to developing comprehensive understanding of mixed-method research in a high-quality, timely, and low-cost manner (84, 85). Skill development labs allow scholars to experiment through rapid cycles of trial and error, testing their ability to create and research evidence-based ADRD nudges in a low-stakes controlled environment (12, 26).

**Table 2:** Agile nudge university syllabus.

Timeframe and Type	Primary ADRD case	Duration	Content
Virtual Bootcamp Day 1 Type: Knowledge Acquisition	The Research Recruitment and Retention Nudge Unit for ADRD Research	6 h	Section 1: Agile Science and Agile mindset. Section 2: Behavioral Economics and Choice Architecture (Nudge) for the ADRD setting. Section 3: Group-based Debriefing with ADRD Faculty.
Virtual Bootcamp Day 2 Type: Knowledge Acquisition	The Aging Brain Care Program	6 h	Section 1: Agile Nudge Innovation, Agile Nudge Implementation, and Agile Nudge Diffusion using ADRD examples in specialty care, primary care, and community-based settings. Section 2: Group-based Debriefing with ADRD Faculty.
Virtual Bootcamp Day 3 Type: Skill Development Lab	Digital Detection of Dementia studies	6 h	Section 1: Self-paced online interactive story simulation module for the Agile Nudge Innovation for the ADRD population in primary care and community-based settings. Section 2: Group-based Debriefing with ADRD Faculty.
Virtual Bootcamp Day 4 Type: Skill Development Lab	The Perioperative Brain Care Program	6 h	Section 1: Self-paced online interactive story simulation module for the Agile Nudge Implementation in inpatient ADRD settings. Section 2: Group-based Debriefing with ADRD Faculty.
Virtual Bootcamp Day 5 Type: Skill Development Lab	Adverse Cognitive Effects of Anticholinergics	6 h	Section 1: Self-paced online interactive story simulation module for the Agile Nudge Diffusion across multiple ADRD settings. Section 2: Group-based Debriefing with ADRD Faculty. Section 3: Online Evaluation Survey.
Virtual Biweekly Feedback Sessions	All case examples will be used	1 h/24 h per scholar per year	On demand one-on-one personalized one-hour nudge coaching session with a dedicated ADRD mentor.
Virtual Monthly Innovation Forums	All case examples will be used	2 h/24 h per year	Group-based problem-solving summits for designing, implementing, or diffusing nudges for use across multiple ADRD settings.
Online ADRD Nudge Resource Library	All case examples will be used	25 h of content	An online open-source resource library of lectures, stories, behavioral and journey mapping tools, minimally specified cognitive biases, minimally specified evidence-based nudges to change individual and organizational behaviors across multiple ADRD settings.

The hour-long biweekly coaching sessions are one-on-one interactions between scholars and interdisciplinary ADRD faculty. Agile Nudge University scholars are expected to engage in nudge research projects throughout the program; therefore, personalized mentorship allows for specific planning and feedback. Prior to mentorship, a mentor-mentee pledge takes place which defines the roles and expectations of the relationship. The minimally standardized operating procedure for Agile mentorship requires sharing crises (drastic situations), noise (personal or professional stressors), positives (something good from the week), and having an open space for questions or discussion (see Table 3). Mentees are also encouraged to explicitly define their primary goal and measure daily progress with a dashboard. The use of Agile Science and the Agile mindset to build effective mentorship relationships has been found to increase mentee performance through quick identification of shortcomings, adoption of an adaptive perspective, better management of relations, and personal growth (86).

**Table 3:** Agile mentorship process minimal standard operating procedure.

Agile Mentorship Process	
At Start	Mentor-Mentee Pledge
	- This is a set of guidelines that define the roles and expectations of mentoring relationships.
Ritual	∼30 min 1:1 meeting. Summary of meeting with reflection. Global Performance Scorecard (GPS) shared with mentor.
Format of Meeting	Mentee shares the following in meeting: • Crises (defined as a personal or professional situation that is paralyzing). • Noise (defined as a personal or professional situation causing mentee stress but not preventing progress). • Positive (defined as something positive that occurred that week). • Discussion(s)/Question(s)
Guidelines	Established psychological safety; nonjudgmental, actionable feedback provided to mentee; defined preferred communication channels (i.e. virtual, in-person, phone call).
Quarterly	Mentor Team Meeting (Primary mentor +3 interdisciplinary members+mentee)
Outcomes	• Define Wildly Important Goal (WIG), leading measures, and lagging measures. • Develop Brand (vision, mission, values, why statements). • Develop skills in Agile Science, emotional intelligence, communication, and networking.
• The Wildly Important Goal (WIG) defined at the start of the mentorship relationship. Progress towards this goal is measured using leading and lagging measures and tracked weekly on a Global Performance Scoreboard (GPS). • Brand development involves the mentee outlining their vision, mission, values, and why statement for use in elevator pitches, introductions, and networking. • Skills in Agile Science involved the application of underlying theories (behavioral economics, complexity science, and network science) to understand, predict, and nudge pro-social behavior of both the overall system and the individual human. • Skills in emotional intelligence include the identification, understanding, and application of emotions to confidently manage communication, conflicts, and anxiety, empathize with others, and problem solve. • Skills in communication included the interpretation and application of nonverbal, verbal, written, open and closed-loop channels. • Skills in discovery including associative thinking, networking, observation, and questioning.	

Expected outcomes of the program include a minimum attendance of 10 participants for each five-day Bootcamp. Innovation Forums are expected to have at least 15 participants, with a minimum of 25% being Agile Nudge University ADRD scientists and 25% being representatives of the target population impacted by the behavioral research project (people living with ADRD, informal caregivers, healthcare delivery system leaders, or healthcare professionals). Net Promoter Scores (NPS) are expected to be over 30 for Bootcamps and Innovation Forums, where an NPS-100-0 needs improvement, 0–30 is good, 30–70 is great, and 70–100 is excellent (87). NPS are a reliable satisfaction metric indicative of loyalty and program scalability (87–89). Each Agile Nudge University ADRD scientist is expected to present a nudge research project in at least one monthly Innovation Forum and is expected to utilize 50% of available biweekly personalized coaching and mentoring sessions. Annually, each Agile Nudge University ADRD scientist is expected to have a minimum of one nudge research project, one publication related to a nudge project, submit one NIH grant application for an ADRD nudge research project, and complete a nudge research project with two or more other Agile Nudge University ADRD collaborators. The open-source resource library is expected to have 100 online users within the first year and an increased number of users for each additional year of the program.

Preliminary data from Cohort 1 shows a total of 14 scholars; 5 of which hold MDs, 7 of which hold PhDs, and 2 of which are in training for PhDs. The first Bootcamp had 20 members in attendance. Across the Innovation Forums, the average NPS was 58 (SD 20.87) with an average of 13 attendees (SD 1.60), 51% from the Agile Nudge University and 28% from the target population. Cohort 2 consists of 13 members, with 2 MDs, 9 PhDs, 1 MBA, and 1 PhD/RN/ACN-BC. Members are spread out across 5 different states and working on nudge projects ranging from scaling local ADRD screening to tailoring interventions to Southern African American ADRD patients to increasing intergenerational interactions for aging populations. The second Bootcamp conducted had 21 attendees. The online resource library was launched in July of 2023, no further data is available at this time.

## 4. Discussion

The Agile Nudge University is committed to providing actionable, high-impact, interdisciplinary training to generate a diverse ADRD workforce competent to design, implement, and sustain nudges that promote high-quality, non-discriminatory, evidence-based care. While Agile Science has been used on a project-based level to increase evidence-based care for aging adults in the past, and other general educational programs for developing competency in Agile methodologies have proved effective, the current program's novel integration of Agile Science and nudge theory specific to ADRD care provide the necessary foundation to overcome structural barriers (12–18, 25). Moreover, Agile Nudge Innovation, Implementation, and Diffusion directly provide steps to facilitate the creation, implementation, and diffusion of evidence-based behavioral ADRD interventions. Already, the program has been shown to have satisfactory enrollment of desired professionals, with a variety of ADRD nudge research projects underway. While Innovation Forum attendance was slightly lower than anticipated, scholars and target populations are adequately represented with “great” NPS scores. Bootcamp attendance has been higher than expected.

Compared with traditional or waterfall project management approaches, Agile methodology is statistically more likely to succeed by a factor of 12%–73% (90). Agile methodology can work in conjunction or independently of other project management strategies – such as lean or Six Sigma – adding responsiveness and adaptability (91). An Agile approach is faster and more human-centered than other methodologies, empowering teams to adjust to changing environmental demands and the target populations' needs (11, 12, 37, 91). Agile methodologies can also be differentiated by not treating individual agents as purely rational and by accounting for organizational complexity within a constantly changing external environment (11, 12, 36, 58–60). By engineering a network of diverse and interdisciplinary scientists, the Agile Nudge University seeks to develop well-versed and interconnected catalysts of change.

Evaluation of the Agile Nudge University will be comprised of both formative and summative components. Formative evaluations will include scholar-led focus groups after Bootcamps and at the end of the academic year to assess the tools, processes, and cumulative experience. Such scholar-driven discussions are known to produce credible data on instructional, mentorship, and content quality (87–89, 92). An ongoing performance review will monitor short-term outcomes through nudge research projects, submitted abstracts, papers, and grants to continuously fine-tune the program. Bootcamp, Innovation Forum, and mentorship evaluations will provide feedback to improve program engagement after each session. Finally, a competency survey will be sent annually to an outside institutional mentor of each participant post-completion requiring them to select if the scholar reached mastery in related categories. Collectively, these formative measures provide feedback to monitor and adjust the program over the course of the minimum 5-year duration. The long-term objectives of the program, namely having developed a sustained interdisciplinary network of scientists able to use nudges to improve ADRD care, will be evaluated at a summative level: total grants and funding within 3 years of completion, career research awards within 3 years of training, and research career status 5 years post-completion. After 5 years, evaluations will track the number of participants who attain promotion, major institutional or national leadership roles, or secure high-level governmental or industry positions. Evaluations will occur through annual post-graduation inquiries about publications, awards, impressions, or career advancements. Given the intention of building a national network of highly engaged ADRD scientists, Agile Nudge University scholars will be encouraged to obtain an ORCID ID, the de facto standard of research disambiguation. By monitoring Scorpus IDs, having automatic alerts on authorships, and using the NIH's RePORTER for grants, a comprehensive view of research accomplishments can be formed.

Limitations to benefits of Agile Nudge University center around logistical constraints. Innovation Forums are limited to a maximum of 25 participants and Bootcamps are limited to 30 scholars to facilitate productive discussions with the involvement of everyone in attendance. Having one-on-one feedback sessions with expert faculty mentors further limit program expansion and scale-up. Cohorts will vary in size depending on recruitment, the number of applications, and applicant fit for the program. The open-source library's impact has not yet been measured but will be assessed by the number of individuals visiting the site and using its resources. Despite an array of formative and summative outcome measurements and targets, the lack of a control group will prevent any formal causal analyses.

## 5. Conclusion

To address growing socioeconomic disparities and burdens associated with ADRD in the United States, diverse interdisciplinary researchers able to resolve translational care gaps will be vital for the success of existing and future research. Through individualized mentoring and mastery of Agile Nudge Innovation, Implementation, and Diffusion, the Agile Nudge University seeks to train scholars to facilitate best practice ADRD research and care in localized social, physical, and digital environments. By itself, the limited scope of such a program is inadequate to overcome the vast barriers in ADRD innovation and implementation research on a national scale. Already, the resource library is an open-source toolkit for using Agile Science to develop evidence-based specific nudges. Broad dissemination of the Agile Nudge University structure and curriculum can be implemented quickly and informed by diffusion theories, offering a way to further expand the frontier in ADRD translation care. By creating champions of the Agile Nudge University and providing full transparency of the program's methodology, increased initiatives in ADRD implementation science and diversity will be necessary to sustain a positive individual, familial, and societal impact.

## Data Availability

The original contributions presented in the study are included in the article/Supplementary Material, further inquiries can be directed to the corresponding author.

## References

[1] National Academies of Sciences, Engineering, and Medicine. Meeting the challenge of caring for persons living with dementia and their care partners and caregivers: A way forward. Washington, DC: The National Academies Press (2021). 10.17226/26026. 33625814

[2] National Academies of Sciences, Engineering, and Medicine. Reducing the impact of dementia in America: A decadal survey of the behavioral and social sciences. Washington, DC: The National Academies Press (2021). 10.17226/26175. 34613684

[3] Sferrazza C Li R, Associates, Inc.,. under contract to the National Institute on Aging, Division of Behavioral and Social Research (NIA/BSR). Virtual Summit Series Summary Report. National Research Summit on Care, Services, and Supports for Persons Living with Dementia and Their Caregivers. National Institute on Aging. National Institutes of Health. July 10, July 21, and August 13, 2020. Available at: https://www.nia.nih.gov/sites/default/files/2021-01/DementiaCareSummitReport.pdf.

[4] Alzheimer’s Association. Alzheimer’s Disease Facts and Figures. 2021; Available at: https://www.alz.org/media/Documents/alzheimers-facts-and-figures-2021-r.pdf (2021).

[5] Matthews KA Xu W Gaglioti AH Holt JB Croft JB Mack D Racial and ethnic estimates of Alzheimer’s disease and related dementias in the United States (2015–2060) in adults aged ≥65 years. Alzheimers Dement. (2019) 15(1):17–24. 10.1016/j.jalz.2018.06.3063 30243772 PMC6333531

[6] Barnes LL. Biomarkers for Alzheimer dementia in diverse racial and ethnic minorities-A public health priority. JAMA Neurol. (2019) 76(3):251–3. 10.1001/jamaneurol.2018.3444 30615027

[7] McDonough IM. Beta-amyloid and cortical thickness reveal racial disparities in preclinical Alzheimer’s disease. Neuroimage Clin. (2017) 16:659–67. 10.1016/j.nicl.2017.09.014 29868439 PMC5984571

[8] Lennon JC Aita SL Bene VAD Rhoads T Resch ZJ Eloi JM Black and white individuals differ in dementia prevalence, risk factors, and symptomatic presentation. Alzheimers Dement. (2022) 18(8):1461–71. 10.1002/alz.12509 34854531 PMC9160212

[9] Castro DM Dillon C Machnicki G Allegri RF. The economic cost of Alzheimer’s disease: family or public health burden? Dement Neuropsychol. (2010) 4(4):262–7. 10.1590/S1980-57642010DN40400003 29213697 PMC5619058

[10] Ho JY Franco Y. The rising burden of Alzheimer’s disease mortality in rural America. SSM Popul Health. (2022) 17:101052. 10.1016/j.ssmph.2022.101052 35242995 PMC8886050

[11] Boustani M Unützer J Leykum LK. Design, implement, and diffuse scalable and sustainable solutions for dementia care. J Am Geriatr Soc. (2021) 69(7):1755–62. 10.1111/jgs.17342 34245584

[12] Boustani M Azar J Solid CA. Agile implementation: A model for implementing evidence-based healthcare solutions into real-world practice to achieve sustainable change. New York City, NY: Morgan James Publishing (2020).

[13] Callahan CM Boustani MA Unverzagt FW Austrom MG Damush TM Perkins AJ Effectiveness of collaborative care for older adults with Alzheimer disease in primary care: a randomized controlled trial. JAMA. (2006) 295(18):2148–57. 10.1001/jama.295.18.2148 16684985

[14] Campbell NL Holden RJ Tang Q Boustani MA Teal E Hillstrom J Multicomponent behavioral intervention to reduce exposure to anticholinergics in primary care older adults. JAGS. (2021) 69(6):1490–9. 10.1111/jgs.17121 PMC8959093 33772749

[15] French DD LaMantia MA Livin LR Herceg D Alder CA Boustani MA. Healthy aging brain center improved care coordination and produced net savings. Health Aff. (2014) 33(4):613–8. 10.1377/hlthaff.2013.1221 24711322

[16] Alder CA Callahan CM Boustani MA Hendrie HC Austrom MG. Providing care to the caregiver: implementing the PREVENT model in a real world memory care clinic. In: Thyrian JR HoffMann W, editors. Dementia care research: Scientific evidence, current issues and future perspectives. Miami, FL: Pabst Science Publishers (2012). p. 34–42.

[17] Boustani MA Sachs GA Alder CA Munger S Schubert CC Guerriero Austrom M Implementing innovative models of dementia care: the healthy aging brain center. Aging Ment Health. (2011) 15(1):13–22. 10.1080/13607863.2010.496445 21271387 PMC3077086

[18] Callahan CM Boustani MA Weiner M Beck RA Livin LR Kellams JJ Implementing dementia care models in primary care settings: the aging brain care medical home. Aging Ment Health*.* (2011);15(1):5–12. 10.1080/13607861003801052 20945236 PMC3030631

[19] Alzheimer’s Association. Alzheimer’s Disease Facts and Figures. 2021; Available at: https://www.alz.org/media/Documents/alzheimers-facts-and-figures-2021-r.pdf (Accessed September 7, 2021) (2021).

[20] Vickrey BG Mittman BS Connor KI Pearson ML Della Penna RD Ganiats TG The effect of a disease management intervention on quality and outcomes of dementia care: a randomized, controlled trial. Ann Intern Med. (2006) 145(10):713–26. 10.7326/0003-4819-145-10-200611210-00004 17116916

[21] Boustani M Alder CA Solid CA Reuben D. An alternative payment model to support widespread use of collaborative dementia care models. Health Aff. (2019) 38(1):54–9. 10.1377/hlthaff.2018.05154 30615525

[22] Boustani MA Frame A Munger S Healey P Westlund J Farlow M Connecting research discovery with care delivery in dementia: the development of the Indianapolis discovery network for dementia. Clinc Interv in Aging. (2012) 7:509–16. 10.2147/CIA.S36078 PMC3508557 23204843

[23] Callahan CM Bateman DR Wang S Boustani MA. State of science: bridging the science-practice gap in aging, dementia and mental health. J Am Geriatr Soc. (2018) 66(Suppl 1):S28–35. 10.1111/jgs.15320 29659003 PMC6690193

[24] Bartlett W. The hidden cost of poor patient engagement in Healthcare. Intelichart. (2022). , Available at: https://www.intelichart.com/blog/the-hidden-cost-of-poor-patient-engagement# (Accessed April 2023)

[25] Mehta J Aalsma MC O’Brien A Boyer TJ Ahmed RA Summanwar D Becoming an Agile change conductor. Front Public Health. (2022) 10:1044702. 10.3389/fpubh.2022.1044702 36589970 PMC9794851

[26] Boustani M Holden RJ Azar J Solid CA. The Agile network: A model to foster innovation, implementation, and diffusion in healthcare systems. Saint Paul, MN: Beaver’s Pond Press (2020).

[27] Holden RJ Boustani MA. The value of an “Agile” mindset in times of crisis. Modern Healthcare. (2020). Available at: https://www.modernhealthcare.com/opinion-editorial/value-agile-mindset-times-crisis.

[28] Chaudoir SR Dugan AG Barr CH. Measuring factors affecting implementation of health innovations: a systematic review of structural, organizational, provider, patient, and innovation level measures. Implement Sci. (2013) 8:22. 10.1186/1748-5908-8-22 23414420 PMC3598720

[29] Holden RJ Carayon P Gurses AP Hoonakker P Hundt AS Ozok AA SEIPS 2.0: a human factors framework for studying and improving the work of healthcare professionals and patients. Ergonomics. (2013) 56(11):1669–86. 10.1080/00140139.2013.838643 24088063 PMC3835697

[30] Stetler CB McQueen L Demakis J Mittman BS. An organizational framework and strategic implementation for system-level change to enhance research-based practice: QUERI series. Implement Sci. (2008) 3:30. 10.1186/1748-5908-3-30 18510750 PMC2430586

[31] Hagedorn H Hogan M Smith JL Bowman C Curran GM Espadas D Lessons learned about implementing research evidence into clinical practice. Experiences from VA QUERI. J Gen Intern Med. (2006) 21(Suppl 2):S21–4. 10.1111/j.1525-1497.2006.00358.x 16637956 PMC2557131

[32] Crabtree BF Nutting PA Miller WL McDaniel RR Stange KC Jaen CR Primary care practice transformation is hard work: insights from a 15-year developmental program of research. Med Care. (2011) 49(Suppl):S28–35. 10.1097/MLR.0b013e3181cad65c 20856145 PMC3043156

[33] Leykum LK Pugh JA Lanham JH Harmon J McDaniel RR. Implementation research design: integrating participatory action research into randomized controlled trials. Implement Sci. (2009) 4:69. 10.1186/1748-5908-4-69 19852784 PMC2770984

[34] Gupta DM Boland RJ Aron DC. The physician’s experience of changing clinical practice: a struggle to unlearn. Implement Sci. (2017) 12:28. 10.1186/s13012-017-0555-2 28245849 PMC5331724

[35] Hanney SR Castle-Clarke S Grant J Guthrie S Henshall C Mestre-Ferrandiz J How long does biomedical research take? Studying the time taken between biomedical and health research and its translation into products, policy, and practice. Health Res Policy Syst. (2015) 13(1):1–18. 10.1186/1478-4505-13-1 25552353 PMC4297458

[36] Thaler RH Sustein CR. Nudge: Improving decisions about health, wealth, and happiness. New York: Yale University Press (2008).

[37] Holden RJ Boustani MA Azar J. Agile innovation to transform healthcare: innovating in complex adaptive systems is an everyday process, not a light bulb event. BMJ Innov. (2021) 7(2):499–505. 10.1136/bmjinnov-2020-000574 PMC13355240 42437033

[38] Boustani M Alder CA Solid CA. Agile implementation: a blueprint for implementing evidence-based healthcare solutions. J Am Geriatr Soc. (2018) 66(7):1372–76. 10.1111/jgs.15283 29513360

[39] Boustani MA van der Marck MA Adams N Azar JM Holden RJ Vollmar HC Developing the agile implementation playbook for integrating evidence-based health care services into clinical practice. Acad Med. (2019) 4:556–61. 10.1097/ACM.0000000000002497

[40] Hekler EB Klasnja P Riley WT Buman MP Huberty J Rivera DE Agile science: creating useful products for behavior change in the real world. Transl Behav Med. (2016) 6(2):317–28. 10.1007/s13142-016-0395-7 27357001 PMC4927453

[41] Braams SM. The software development landscape: a rationalization of agile software development as a strategy in the face of organizational complexity [Unschede, The Netherlands: behavioural, management and social sciences. University of Twente (2020).

[42] Lopez-Alcarria A Olivares-Vicente A Poza-Vilches F. A systematic review of the use of Agile methodologies in education to foster sustainability competencies. Sustain. (2019) 11(10):2915. 10.3390/su11102915

[43] Khan BA Lasiter S Boustani MA. CE: critical care recovery center: an innovative collaborative care model for ICU survivors. Am J Nurs. (2015) 115(3):24–31; quiz 34, 46. 10.1097/01.NAJ.0000461807.42226.3e 25674682 PMC4608259

[44] Holden RJ Srinivas P Campbell NL Clark DO Bodke KS Hong Y Understanding older adults’ medication decision making and behavior: a study on over-the-counter (OTC) anticholinergic medications. RSAP. (2019) 15(1):53–60. 10.1016/j.sapharm.2018.03.002 29559218 PMC6690198

[45] Campbell NL Perkins AJ Khan BA Gao S Farber MO Khan S Deprescribing in the pharmacologic management of delirium: a randomized trial in the intensive care unit. J Am Geriatr Soc. (2019) 67(4):695–702. 10.1111/jgs.15751 30664239 PMC6540083

[46] Azar J Kelley K Dunscomb J Perkins A Wang Y Beeler C Using the Agile implementation model to reduce central line-associated bloodstream infections. Am J of Infect Control. (2019) 47(1):33–7. 10.1016/j.ajic.2018.07.008 30201414

[47] Braly T Muriathiri D Brown JC Taylor BM Boustani MA Holden RJ. Technology intervention to support caregiving for Alzheimer’s disease (I-CARE): study protocol for a randomized controlled pilot trial. Pilot Feasibility Stud. (2021) 7(1):23. 10.1186/s40814-020-00755-2 33431027 PMC7798342

[48] Fowler NR Head KJ Perkins AJ Gao S Callahan CM Bakas T Examining the benefits and harms of Alzheimer’s disease screening for family members of older adults: study protocol for a randomized controlled trial. Trials. (2020) 21(1):202. 10.1186/s13063-019-4029-5 32075686 PMC7031904

[49] Fowler NR Perkins AJ Gao S Sachs GA Boustani MA. Risks and benefits of screening for dementia in primary care: the Indiana university cognitive health outcomes investigation of the comparative effectiveness of dementia screening (IU CHOICE) *Trial*. J Am Geriatr Soc. (2020) 68(3):535–43. 10.1111/jgs.16247 31792940 PMC7187902

[50] Wang S Hanneman P Xu C Gao S Allen D Golovyan D Critical care recovery center: a model of agile implementation in intensive care unit (ICU) survivors. Int Psychogeriatr. (2020) 32(12):1409–18. 10.1017/S1041610219000553 31466536 PMC7048643

[51] Holden RJ Campbell NL Abebe E Clark DO Ferguson D Bodke K Usability and feasibility of consumer-facing technology to reduce unsafe medication use by older adults. Res Social Adm Pharm. (2020) 16(1):54–61. 10.1016/j.sapharm.2019.02.011 30853507 PMC6710164

[52] Khan BA Perkins AJ Campbell NL Gao S Farber MO Wang S Pharmacological management of delirium in the intensive care unit: a randomized pragmatic clinical trial. J Am Geriatr Soc. (2019) 67(5):1057–65. 10.1111/jgs.15781 30681720 PMC6492267

[53] Wang S Hammes J Khan S Gao S Harrawood A Martinez S Improving recovery and outcomes every day after the ICU (IMPROVE): study protocol for a randomized controlled trial. Trials. (2018) 19(1):196. 10.1186/s13063-018-2569-8 29580264 PMC5869765

[54] Khan S Biju A Wang S Gao S Irfan O Harrawood A Mobile critical care recovery program (m-CCRP) for acute respiratory failure survivors: study protocol for a randomized controlled trial. Trials. (2018) 19(1):94. 10.1186/s13063-018-2449-2 29415760 PMC5803999

[55] Leykum LK Lanham HJ Pugh JA Parchman M Anderson RA Crabtree BF Manifestations and implications of uncertainty for improving healthcare systems: an analysis of observational and interventional studies grounded in complexity science. Implement Sci. (2014) 9:165. 10.1186/s13012-014-0165-1 25407138 PMC4239371

[56] Anderson RA Crabtree BF Steele DJ McDaniel RR Jr. Case study research: the view from complexity science. Qual Health Res. (2005) 15(5):669–85. 10.1177/1049732305275208 15802542 PMC1822534

[57] Boustani MA Munger S Gulati R Vogel M Beck RA Callahan CM. Selecting a change and evaluating its impact on the performance of a complex adaptive health care delivery system. Clin Interv Aging. (2010) 5:141–8. 10.2147/CIA.S9922 20517483 PMC2877524

[58] Holland JH. Signals and boundaries: Building blocks for complex adaptive systems. Cambridge, MA: MIT Press (2012).

[59] Chaiken S Trope Y. Dual-process theories in social psychology. New York: Guilford Press (1999).

[60] Kahneman D. Thinking, fast and slow. New York: Farrar, Straus and Giroux (2011).

[61] Patel MS Volpp KG Asch DA. Nudge units to improve the delivery of health care. N Engl J Med. (2018) 378(3):214–6. 10.1056/NEJMp1712984 29342387 PMC6143141

[62] Mrkva K Posner NA Reeck C Johnson EJ. Do nudges reduce disparities? Choice architecture compensates for low consumer knowledge. J Mark. (2021) 85(4):67–84. 10.1177/0022242921993186

[63] Yoong SL Hall A Stacey F Grady A Sutherland R Wyse R Nudge strategies to improve healthcare providers’ implementation of evidence-based guidelines, policies and practices: a systematic review of trials included within cochrane systematic reviews. Implement Sci. (2020) 15(1):50. 10.1186/s13012-020-01011-0 32611354 PMC7329401

[64] Shachar T Greenbaum D. When a push becomes a shove: nudging in elderly care. Am J Bioeth. (2019) 19(5):78–80. 10.1080/15265161.2019.1588415 31090528

[65] Last BS Buttenheim AM Timon CE Mitra N Beidas RS. Systematic review of clinician-directed nudges in healthcare contexts. BMJ Open. (2021) 11(7):e048801. 10.1136/bmjopen-2021-048801 34253672 PMC8276299

[66] Hummel D Maedche A. How effective is nudging? A quantitative review on the effect sizes and limits of empirical nudging studies. J Behav Exp Econ. (2019) 80:47–58. 10.1016/j.socec.2019.03.005

[67] Cammisuli DM Pietrabissa G Castelnuovo G. Improving wellbeing of community-dwelling people with mild cognitive impairment: the SENIOR (SystEm of nudge theory based ICT applications for OldeR citizens) project. Neural Regen Res. (2021) 16(5):963–6. 10.4103/1673-5374.297063 33229736 PMC8178777

[68] Barabasi AL. Network science. New York, NY: Cambridge University Press (2016).

[69] Rogers EM. Diffusion of innovations. 5th ed New York: Free Press (2003).

[70] Rogers EM Medina UE Rivera MA Wiley CJ. Complex adaptive systems and the diffusion of innovations. J Public Sect Innov. (2005) 10(3).

[71] Greenhalgh T Robert G Macfarlane F Bate P Kyriakidou O. Diffusion of innovations in service organizations: systematic review and recommendations. Milbank Q. (2004) 82(4):581–629. 10.1111/j.0887-378X.2004.00325.x 15595944 PMC2690184

[72] West G. Scaling dynamics. Penguin Press (2017). Available at: http://robdunnlab.com/projects/beats-per-life/.

[73] Centola D. Change: How to make big things happen. New York: Little, Brown Spark (2021). ISBN 978-1-529-37338-7.

[74] Williams J Kolb HR. Communication in clinical research: uncertainty, stress, and emotional labor. J Clin Transl Sci. (2021) 6(1):e11. 10.1017/cts.2021.873 35211337 PMC8826006

[75] Chen Y Yu C Yuan Y Lu F Shen W. The influence of trust on creativity: a review. Front Psychol. (2021) 12:706234. 10.3389/fpsyg.2021.706234 34484060 PMC8415111

[76] Mitton C Peacock S Storch J Smith N Cornelissen E. Moral distress among healthcare managers: conditions, consequences and potential responses. Healthc Policy. (2010) 6(2):99–112. 10.12927/hcpol.2010.22036 22043226 PMC3016638

[77] Wolor CW Ardiansyah A Rofaida R Nurkhin A Rababah MA. Impact of toxic leadership on employee performance. Health Psychol Res. (2022) 10(4):57551. 10.52965/001c.57551 36540087 PMC9760724

[78] Vermeir P Vandijck D Degroote S Peleman R Verhaeghe R Mortier E Communication in healthcare: a narrative review of the literature and practical recommendations. Int J Clin Pract. (2015) 69(11):1257–67. 10.1111/ijcp.12686 26147310 PMC4758389

[79] Tiwary A Rimal A Paudyal B Sigdel KR Basnyat B. Poor communication by health care professionals may lead to life-threatening complications: examples from two case reports. Wellcome Open Res. (2019) 4:7. 10.12688/wellcomeopenres.15042.1 31448336 PMC6694717

[80] Glanz K Bishop DB. The role of behavioral science theory in development and implementation of public health interventions. Annu Rev Public Health. (2010) 31:399–418. 10.1146/annurev.publhealth.012809.103604 20070207

[81] National Institute on Aging. Health disaparity framework. Available at: https://www.nia.nih.gov/research/osp/framework.

[82] Agile Nudge University Program. 2023. Available at: https://hii.iu.edu/education/agile-nudge-university.html.

[83] Spagnoli L Comte E Sheath D Rosset N Loutan L Geissbuhler A Geneva Health forum: the forum of innovative practices in global health. Int J Environ Res Public Health. (2020) 17(5):1517. 10.3390/ijerph17051517 32120869 PMC7084837

[84] Guise JM Hansen M Lambert W O’Brien K. The role of simulation in mixed-methods research: a framework & application to patient safety. BMC Health Serv Res. (2017) 17(1):322. 10.1186/s12913-017-2255-7 28472958 PMC5418848

[85] Guo C Ashrafian H Ghafur S Fontana G Gardner C Prime M. Challenges for the evaluation of digital health solutions-A call for innovative evidence generation approaches. NPJ Digit Med. (2020) 3:110. 10.1038/s41746-020-00314-2 32904379 PMC7453198

[86] Lindroth H Shumaker C Taylor B Boiustani Z Boustani M. Agile mentorship: a longitudinal exploratory analysis. ATS Scholar Journal. (2023) 4(2):132–44. 10.34197/ats-scholar.2022-0035PS PMC10394690 37538074

[87] Grigore. What is a good net promoter score? (2022 NPS benchmark). Retently. Retrieved June 27, 2022, Available at: https://www.retently.com/blog/good-net-promoter-score/ (2022).

[88] Reichheld FF. The one number you need to grow. Harv Bus Rev. (2003) 81(12):46–124. 14712543

[89] Reichheld Frederick F Rob M. The ultimate question 2.0: How net promoter companies thrive in a customer-driven world. Boston, MA: Harvard Business Review Press (2011).

[90] Nordmark S Lindberg I Zingmark K. “It’s all about time and timing”: nursing staffs’ experiences with an agile development process, from its initial requirements to the deployment of its outcome of ICT solutions to support discharge planning. BMC Med Inform Decis Mak. (2022) 22(1):186. 10.1186/s12911-022-01932-4 35843948 PMC9288650

[91] Improta G Guizzi G Ricciardi C Giordano V Ponsiglione AM Converso G Agile six sigma in healthcare: case study at santobono pediatric hospital. Int J Environ Res Public Health. (2020) 17(3):1052. 10.3390/ijerph17031052 32046052 PMC7037742

[92] Bamberger M Rugh J Mabry L. Real world evaluation: working under budget, time, data and political constraints. Thousand Oaks, CA: Sage Publications (2006).

